# Hepatocyte growth factor modulates motility and invasiveness of ovarian carcinomas via Ras-mediated pathway

**DOI:** 10.1054/bjoc.1999.1016

**Published:** 2000-02

**Authors:** Y Ueoka, K Kato, Y Kuriaki, S Horiuchi, Y Terao, J Nishida, H Ueno, N Wake

**Affiliations:** Department of Reproductive Physiology and Endocrinology, Medical Institute of Bioregulation, Kyushu University 4546 Tsurumihara, Beppu, Oita, 874-0838, Japan; Department of Cardiology, Kyushu University, Fukuoka, Japan

**Keywords:** hepatocyte growth factor, motility, invasion, ovarian carcinoma, *ras*, MAP kinase

## Abstract

Hepatocyte growth factor (HGF) is a multifunctional growth factor which has pleiotrophic biological effects on epithelial cells such as proliferation, motogenesis, invasiveness and morphogenesis. Peritoneal dissemination is critical for the progression of ovarian cancer, and our study revealed that HGF induces migration and invasion of ovarian cancer cells. We also demonstrated that HGF stimulates autophosphorylation of its receptor, followed by activation of the Ras-MAP (mitogen-activated peptide) kinase cascade. Moreover, infection of ovarian cancer cells with Ras dominant-negative adenovirus reduced the HGF-induced motogenic and invasive activities. Additionally, both MEK and PI3-kinase pathways downstream of Ras were involved in HGF-stimulated ovarian cancer cell invasiveness. © 2000 Cancer Research Campaign


*British Journal of Cancer* (2000) **82**(4), 891–899
© 2000 Cancer Research Campaign
DOI: 10.1054/ bjoc.1999.1016, available online at http://www.idealibrary.com on IDEAL®

### Hepatocyte growth factor modulates motility and invasiveness of ovarian carcinomas via Ras-mediated pathway

**Y Ueoka[1], K Kato[1], Y Kuriaki[1], S Horiuchi[1], Y Terao[1], J Nishida[1], H Ueno[2] and N Wake[1]**

[1]Department of Reproductive Physiology and Endocrinology, Medical Institute of Bioregulation, Kyushu University 4546 Tsurumihara, Beppu, Oita, 874-0838, Japan; [2]Department of Cardiology, Kyushu University, Fukuoka, Japan

**Summary** Hepatocyte growth factor (HGF) is a multifunctional growth factor which has pleiotrophic biological effects on epithelial cells such as proliferation, motogenesis, invasiveness and morphogenesis. Peritoneal dissemination is critical for the progression of ovarian cancer, and our study revealed that HGF induces migration and invasion of ovarian cancer cells. We also demonstrated that HGF stimulates autophosphorylation of its receptor, followed by activation of the Ras-MAP (mitogen-activated peptide) kinase cascade. Moreover, infection of ovarian cancer cells with Ras dominant-negative adenovirus reduced the HGF-induced motogenic and invasive activities. Additionally, both MEK and PI3-kinase pathways downstream of Ras were involved in HGF-stimulated ovarian cancer cell invasiveness. © 2000 Cancer Research Campaign

**Keywords:** hepatocyte growth factor; motility; invasion; ovarian carcinoma; *ras*; MAP kinase

The prognosis of ovarian cancers remains poor as approximately two-thirds of the patients have already suffered from stage III or IV disease at the time of diagnosis. Ovarian cancers initially grow locally and invade the capsule. The cancer cells then exfoliate into the peritoneal cavity and disseminate. The patients often have massive ascites involving various kinds of growth factors, suggesting the potential involvement of growth factors in ascites in the progression and dissemination of ovarian cancers. Activation of the signalling pathways mediated by growth factors may contribute to the unrestricted growth that is characteristic of these tumours. Growth factors are also able to stimulate changes in cellular morphology, motility and invasiveness, which may promote both the metastasis and dissemination of tumour cells. Among these growth factors, hepatocyte growth factor (HGF), independently identified as scatter factor, is known to have mitogenic, motogenic and morphogenic actions on a variety of epithelial and endothelial cell types that express its receptor (HGFR) (Stoker et al, 1987; Montesano et al, 1991; Lamszus et al, 1998). The HGF-HGFR system is considered to be a principal paracrine mediator of normal mesenchymal–epithelial interactions (Rosen et al, 1994), and has been also shown to be involved in the formation and spreading of tumours (Jeffer et al, 1996*a*; Nakamura et al, 1997). HGFR is a 190 kDa heterodimer composed of a 50 kDa extracellular α-subunit and a 145 kDa β-subunit. These subunits are synthesized as a single-chain precursor, pro-HGFR. The β-subunit contains the extracellular ligand binding domain, transmembrane domain and intracellular tyrosine kinase domain. Ligand binding to HGFR induces dimerization of adjacent receptor molecules and autophosphorylation in its C-termini. The HGFR tyrosine kinase functions through a Src homology 2 (SH2) docking site that can activate several signalling pathways (Ponzetto et al, 1994; Pellici et al, 1995). The pathway involved in the response to HGF has been tentatively described as follows: the first phase of the response (motogenesis), which results from cytoskeletal reorganization, loss of intercellular junctions and cell migration, is mainly dependent on phosphatidylinositol 3-kinase (PI3-kinase) and Rac activation; the second phase (mitogenesis) requires stimulation of the Ras-MAP kinase (MAPK) cascade; the third phase (morphogenesis) is dependent on the signal transducers and activators of the transcription (STAT) pathway (Boccacino et al, 1998).

There is evidence that changes in the HGF-HGFR system are involved in the development or progression of ovarian cancers. HGFR is expressed in the normal ovarian surface epithelium and various types of primary ovarian cancers (Di Renzo et al, 1994; Moghul et al, 1994). Marked overexpression of HGFR protein occurs in some fractions of ovarian cancers, as determined by Western blotting (Di Renzo et al, 1994; Corps et al, 1997). This suggests that ovarian cancers progress and metastasize to the surrounding tissues through activation of the HGF-HGFR system.

HGF activates Ras protein by shifting the equilibrium toward the guanosine 5′-triphosphate (GTP)-bound state and increases the uptake of guanine nucleotides of Ras (Hartmann et al, 1994; Ridley et al, 1995). We are interested in the Ras-MAPK cascade downstream of HGFR since mutated forms of the human *ras* genes have been detected in a fraction of ovarian cancers (Teneriello et al, 1993). The contribution of this abberant Ras function to mutant Ras-positive tumours remains to be determined, while it is clear that ovarian cancer development can occur in the absence of mutations in *ras* genes. An investigation of physiologically controlled Ras-MAPK signalling in response to HGF would help to determine the role of the abberant Ras function in cancer development. To clarify the role of Ras activation by HGF, we collected a panel of human ovarian cancer cell lines that were able to transmit the HGF-mediated stimuli to MAPK protein through HGFR autophosphorylation. These cells overexpressed HGFR protein and carried the wild-type sequences of the K-, H- and N-*ras* genes. The motility was enhanced by HGF treatment in ovarian cancer cells

*Received 17 February 1999*
*Revised 16 August 1999*
*Accepted 21 September 1999*

*Correspondence to*: N Wake and this presumably occurred concomitantly with the promotion of chemoattraction and the resultant invasiveness. The H-Ras Y57, a dominant-negative Ras protein, abrogated the stimulation of cell migration and invasiveness of the ovarian cancer cells in response to HGF, suggesting that the Ras-MAPK cascade is essential for the motogenic activity of ovarian cancer cells. In addition, the MAPK and the PI3-kinase pathways downstream of Ras are involved in the invasiveness of the cells.

### MATERIALS AND METHODS

### Cells and cell culture

Eight human ovarian cancer cell lines were used in the present study. We obtained KF and MH cells from Dr Toshihiro Kikuchi, National Defense Medical College; MCAS, TYK-nu, Kuramochi, and PA-1 cells from the Health Science Research Resources Bank (HSRRB); HAC-2 cells from Dr Masato Nishida, Tsukuba University; SKOV-3 cells from Dr Hiroaki Kobayashi, Kyushu University. All cell lines were maintained in RPMI medium (Gibco) supplemented with 10% fetal calf serum (FCS; Medical Link).

### Measurement of HGF in culture media

Cells were incubated to confluency in RPMI with 10% FCS, then the medium was replaced with serum-free medium followed by a further incubation for 48 h at 37°C. HGF in the medium was determined by the enzyme-linked immunosorbent assay (ELISA) (IMMUNIS EIA kit, Tokushumeneki Kenkyusho, Japan) according to the manufacturer's instructions.

### Western blotting to determine HGFR expression

HGF receptor expression was analysed by Western blotting. Subconfluent cells were lysed with ice-cold lysis buffer (sodium chloride (NaCl) 100 mM, Tris–HCl pH 7.8, 20 mM, sodium fluoride (NaF) 50 mM, NP-40 1%, glycerol 10%, sodium orthovanadate 1 mM, phenylmethylsulphonyl fluoride 1.25 mM, leupeptin 10 μg ml$^{-1}$, aprotinin 10 μg ml$^{-1}$), and centrifuged at 13 000 ***g*** for 10 min at 4°C to remove debris. The protein concentration was determined using a Coomassie Protein Assay (PIERCE) and 100 μg of each protein was resolved by 10% sodium dodecyl sulphate polyacrylamide gel electrophoresis (SDS-PAGE) and transferred onto a nitrocellulose membrane in a semi-dry transfer cell (Bio Rad). The blots were incubated with the anti-c-Met monoclonal antibody, C-28 (Santa Cruz Biotechnology, Inc), followed by incubation with horseradish peroxidase-linked anti-rabbit antibodies (Amersham), and analysed with ECL system (Amersham).

### Analysis of phosphorylated c-Met

Subconfluent cells were incubated with serum-free medium over night and 100 ng ml$^{-1}$ of HGF was added for 10 min. The cells were lysed on ice in PY buffer (Tris–HCl pH 7.8 20 mM, NaCl 50 mM, NaF 50 mM, $Na_4P_2O_7$ 30 mM, EGTA 5 mM, Triton X-100 1%, sodium orthovanadate 1 mM) containing freshly added protease inhibitors (phenylmethylsulphonyl fluoride 1 mM, leupeptin, 1 μg ml$^{-1}$, aprotinin 10 μg ml$^{-1}$) (Pelicci et al, 1995). One milligram of each lysate was incubated with agarose conjugated anti-c-Met antibody, C-28 (Santa Cruz Biotechnology, Inc.) over night at 4°C. Immunocomplexes were washed with the same buffer twice, resuspended in SDS boiling buffer and resolved by 10% SDS-PAGE. The separated proteins were blotted onto nitrocellulose membranes and the membranes were incubated with the anti-phosphotyrosine antibody, PY99 (Santa Cruz Biotechnology, Inc), followed by incubation with horseradish peroxidase-linked anti-mouse antibodies (Amersham), and analysed with ECL system (Amersham).

### Screening for *ras* mutation

Total RNA from the cells was isolated using ISOGEN (Nippon Gene). One microgram of total RNA was reverse transcribed into cDNA by reverse transcriptase (Perkin-Elmer) and selective amplification of the K-, H- and N-*ras* gene sequence was performed using the polymerase chain reaction (PCR). Amplified DNA fragments were dot-blotted onto a nylon membrane (Hybond-N$^+$, Amersham) and denatured in 0.4 N sodium hydroxide (NaOH). The membrane was pre-hybridized for 1 h at 42°C in hybridization solution (Rapid-hyb buffer, Amersham). Hybridization was performed for 2 h at 42°C in the same solution with 10 pmol $^{32}$P-labelled mutation-specific oligonucleotide probes surrounding codon 12, 13 and 61 of K-, H- and N-ras (Takara). After hybridization, the membrane was washed twice in 1 × SSC (saline-sodium citrate), 0.1% SDS at room temperature for 10 min, and in 0.1 × SSC, 0.1% SDS at 50°C for 20 min. The membrane was exposed to the plate of a Bio-image analyser (BAS1000, FUJIX, Japan). Point mutations were confirmed by sequencing (Takara Taq Cycle Sequencing Kit, Takara) according to the manufacturer's instructions.

### Analysis of MAPK activity

Subconfluent cells were incubated with serum-free RPMI for 48 h and 10 ng ml$^{-1}$ of HGF was added for 15 min. Cells were lysed with lysis buffer (Tris–HCl pH 8.0 20 mM, Triton X-100 1%, glycerol 10%, NaCl 137 mM, magnesium chloride 1.5 mM, EGTA 1 mM, NaF 50 mM, sodium orthovanadate 1 mM) containing freshly added protease inhibitors (phenylmethylsulphonyl fluoride 1 mM, leupeptin 1 μg ml$^{-1}$, aprotinin 10 μg ml$^{-1}$). After centrifugation at 13 000 ***g*** for 10 min to remove debris, 100 μg of the proteins was separated by 12.5% SDS-PAGE and the separated proteins were transferred to nitrocellulose membranes. The membranes were incubated with the MAPK monoclonal antibody, Ab-1 (CalBiochem) or phospho-specific MAPK antibody, pTEpY (Promega) followed by incubation with $^{125}$I-labelled antibody (Amersham). The amount of each protein was quantitated using a Bio-image analyser (BAS1000, FUJIX, Japan).

### Boyden chamber assay

Cell motility was determined using Boyden chambers with 8-μm pore size polycarbonate filters (Coster). NIH3T3 cell-conditioned medium was placed in the lower compartment of the Boyden chambers as a source of chemoattractant. Cells were trypsinized, and $5 \times 10^4$ cells were suspended in 100 μl of RPMI containing 0.1% bovine serum albumin (BSA) and placed in the upper compartment. For the motility assay, 1 or 10 ng ml$^{-1}$ of HGF was added to the lower compartment. For checkerboard analysis, 1 or

 © 2000 Cancer Research Campaign

10 ng ml$^{-1}$ of HGF was added to the upper and/or lower compartment. The cells were incubated for 10 h at 37°C, fixed with methanol, and then stained with haematoxylin and eosin. Cells on the upper surface of the filter were removed with a cotton swab, and cells that had migrated to the lower surface were counted under a light microscope at 200 × magnification. Six fields were counted for each assay and compared to the control.

The invasion assay was performed essentially according to the method reported previously (Kobayashi et al, 1994). The filter was coated with 20 μg of Matrigel (Becton Dickinson Collaborative Research, Bedford, MA, USA), which is a mixture of basement membrane components such as fibronectin, type IV collagen, laminin, vitronectin and proteoglycans. The cells were trypsinized, and $1 \times 10^5$ cells were suspended in 100 μl RPMI containing 0.1% BSA and placed in the upper compartment. NIH3T3 cell-conditioned medium was placed in the lower compartment and incubated for 24 h at 37°C, after which 10 ng ml$^{-1}$ of HGF was added into upper and lower compartments. The subsequent procedures were the same as those of the cell motility assay.

In order to examine whether NIH3T3 cells conditioned medium effects on human Met, Boyden chamber assay was performed with the addition of 500 ng ml$^{-1}$ anti-human HGF antibody, H0652 (SIGMA) into both compartments of the chamber. The same concentration of goat IgG, sc-2028 (Santa Cruz) was used as a control study.

### Adenovirus-mediated gene transfer of dominant negative Ras

A recombinant adenovirus, AdexCAHRasY57, expressing a dominant-negative Ras was generated by cloning the human H-*ras* cDNA, with asparatic acid to tyrosine substitution at amino acid position 57 (Ueno et al, 1997). We also used a control adenovirus, AdLacZ in which lacZ was driven by the SRα promotor and the lacZ expression was evaluated by histostaining.

Co-infection of the cells with adenoviral vectors was carried out by incubating subconfluent cells with vectors in phosphate buffer saline (PBS) for 2 h at room temparature. The cells were then washed with PBS twice and the medium was changed for RPMI containing 10% FCS. The cells were incubated at 37°C until each assay as described.

For the analysis of cell growth, after 24 h of infection, cells were plated at $1 \times 10^4$ cells per 2.0 mm 24-well plates in RPMI supplemented with 10% FCS and incubated for 24 h. Viable cells were counted and compared to the control.

For the motility and invasion assays, $5 \times 10^4$ cells and $1 \times 10^5$ cells, respectively, were plated in a Boyden chamber after 24 h of infection and the Boyden chamber assay was performed as described above.

For the detection of MAPK activity, subconfluent cells were infected with the adenoviral vectors. After 24 h, the medium was replaced with serum-free RPMI for 48 h and 10 ng ml$^{-1}$ HGF was added for 15 min. Cells were lysed and MAPK activity was determined as described above.

### Effect of PD98059 and LY294002 on motility and invasiveness

We used PD98059 and LY294002 as specific inhibitors of MAP kinase kinase (MEK) and PI3-kinase respectively. After 24 h incubation with serum-free medium, subconfluent Kuramochi cells were treated either with 10 μM PD98059 (Cal Biochem) or 50 μM LY294002 (Cal Biochem) dissolved in dimethyl sulphoxide (DMSO). After an additional 24 h incubation, 10 ng ml$^{-1}$ of HGF was added to the culture medium for 15 min, and the cells were lysed. MAP kinase activity was determined for PD98059-treated Kuramochi cells as described above. Proteins (100 μg) from LY294002-treated Kuramochi cells were separated by 10% SDS-PAGE and transferred to nitrocellulose membranes. Each membrane was incubated with the phospho-Akt antibody (Ser473) (New England Biolabs. Inc), followed by incubation with $^{125}$I-labelled antibody (Amersham). The amount of protein was quantified using a Bio-image analyser (BAS1000, Fujix).

For the Boyden chamber assay, Kuramochi cells were plated onto the upper chamber. Either 10 μM PD98059 or 50 μM LY249002 was put in both compartments of the chamber. We used the same concentration of DMSO as a control.

### RESULTS

### HGF-mediated signalling cascade in ovarian cancer cells

In the present study, we examined the role of the Ras-mediated signalling cascade in regulating the potent motogenic activity of HGF in ovarian cancer cells. Thus, we addressed whether the panel of eight ovarian cancer cell lines used here maintained the response to exogenous HGF and HGF-mediated signal transduction. First, we examined the cell lines for production of HGF into the culture media by the ELISA method to exclude the possible autocrine-growth factor pathway. Detectable levels of HGF were absent in the conditioned media from these eight ovarian cancer cell lines, thereby providing no evidence for HGF-HGFR autocrine activity (data not shown).

We determined the relative levels of HGFR expression by Western blotting analysis using a polyclonal anti-peptide antibody directed against the COOH-terminus of the human HGFR (C-28). As shown in Figure 1, high levels of 145 kDa HGFR protein, along with variable levels of its unprocessed precursor (170 kDa) were detected in six cell lines (HAC-2, SKOV-3, Kuramochi, MH, KF and MCAS). Lower but still detectable levels of HGFR were observed in the remaining PA-1 and TYK-nu cells. These observations were in agreement with previous reports of overexpression of HGFR protein in ovarian cancers, across multiple types of tumours, even though the expression level showed substantial variation between tumours (Di Renzo et al, 1994).

Binding of HGFR to its ligand induces autophosphorylation at tyrosine residues and initiates a cascade of intracellular events. To test whether the HGFR overexpressed in these cell lines was tyrosine-phosphorylated in response to HGF, cell lysates were immunoprecipitated with an anti-HGFR antibody (C-28) and immunoblotted with an anti-phosphotyrosine antibody (Figure 2A). HGFR of each cell line was phosphorylated in response to HGF.

We next evaluated the status of ras gene mutations in the panel of cell lines. Dot blot hybridization with mutation-specific oligomers was used to screen for mis-sense mutations at codons 12, 13 and 61 of the K-, H- and N-*ras* genes and further direct sequence analysis was performed on PCR-amplified DNA isolated from each of the cell lines. Two cell lines had a *ras* gene mutation

© 2000 Cancer Research Campaign 

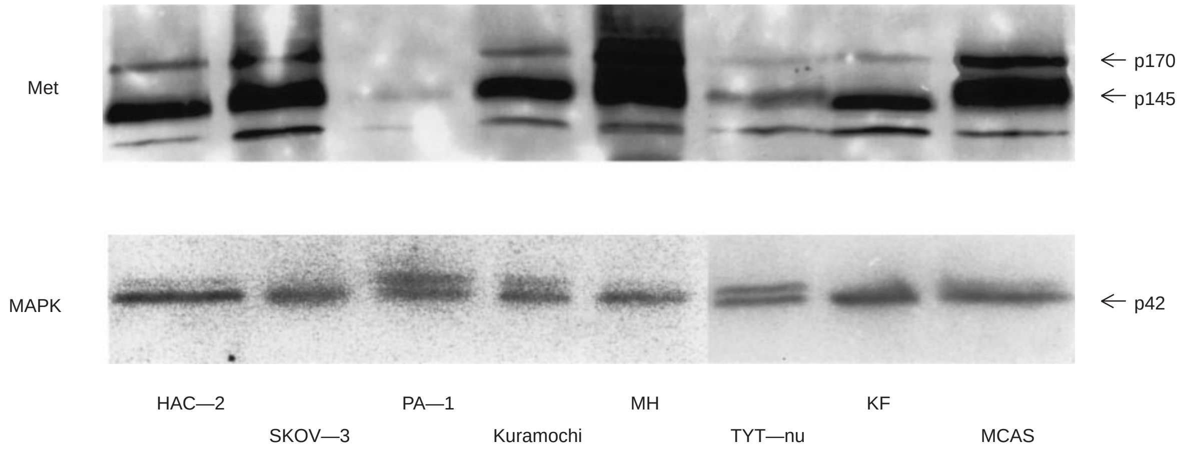

**Figure 1** Western blotting analysis of the HGF receptor. One hundred micrograms of proteins obtained by lysis of each ovarian cancer cell line were analysed. Blots were incubated with monoclonal antibodies the COOH terminal tail of the HGF receptor, which labelled the p145 β-chain and the p170 precursor of the $M_r$ 190 000 receptor, followed by incubation with horseradish peroxidase linked anti-rabbit antibodies (Amersham), and were analysed with ECL system (Amersham). MAPK was shown as an internal control in the lower panel. The arrows indicated the p170 precursor form and p145 β-chain of HGF receptor and p42 MAPK respectively

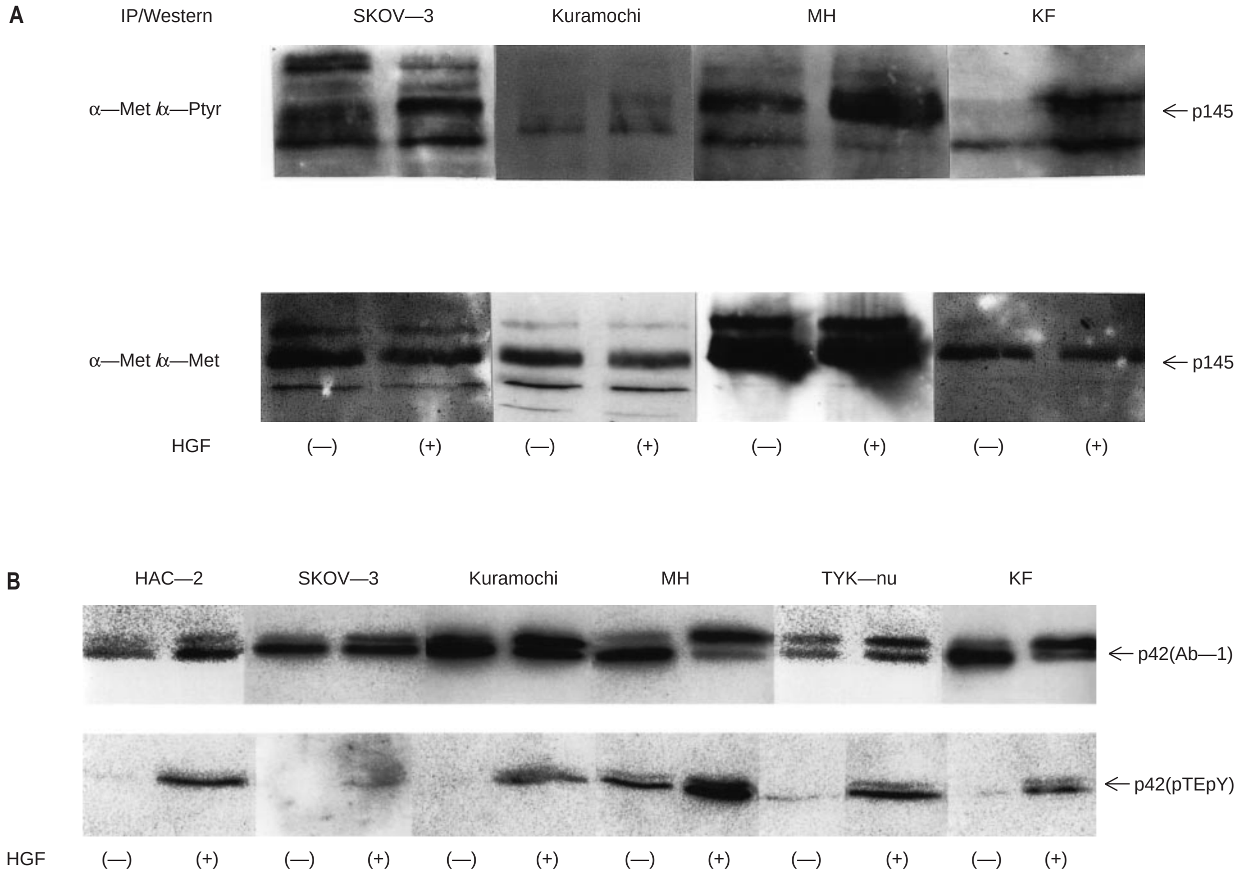

**Figure 2** (**A**) Tyrosine phosphorylation of HGF receptor. Subconfluent cells were incubated with serum-free RPMI for 24 h and 100 ng ml$^{-1}$ of HGF was added for 10 min. Protein extracted by non-ionic detergent in the presence of kinase and phosphatase inhibitors was immunoprecipitated with agarose-conjugated anti-c-Met antibody, separated under reducing conditions, blotted onto nitrocellulose membranes, and probed with anti-phosphotyrosine monoclonal antibody, followed by incubation with horseradish peroxidase-linked anti-rabbit antibodies, and analysed with ECL system. HGF receptor of each cell-line was phosphorylated in response to HGF. The arrow indicates the p145 β-chain of HGF receptor. (**B**) Activation of MAPK by HGF stimulation. Subconfluent cells were incubated with serum-free RPMI for
48 hours and 10 ng ml$^{-1}$ of HGF was added for 15 min. Cell extracts were electrophoresed in polyacrylamide gels and transferred onto nitrocellulose membranes. The blots were incubated with anti-MAPK monoclonal antibody (Ab-1), followed by incubation with $^{125}$I-goat anti-mouse antibody. The upper band of the doublet appearing at 42 kDa represents a hyperphosphorylated form of p42 MAPK. The blots were also incubated with anti-phospho-specific MAPK antibody (pTEpY), followed by incubation with $^{125}$I-goat anti-rabbit antibody. HGF treatment activated MAPK in each cell line

 *© 2000 Cancer Research Campaign*

**Table 1** Invasion assay

| Cell line | | HAC-2 | SKOV-3 | Kuramochi | MH | TYK-nu | KF | Kuramochi +anti-HGF |
|---|---|---|---|---|---|---|---|---|
| Invaded cells/6HPF | HGF (–) | 217 ± 42 | 1424 ± 301 | 134 ± 71 | 200 ± 81 | 808 ± 85 | 452 ± 83 | 163 ± 18 |
| | HGF(+) | 195 ± 30 | 2309 ± 110 | 678 ± 188 | 670 ± 126 | 1503 ± 113 | 999 ± 223 | 204 ± 29 |
| Activation of invasion (fold) | | 0.90 | 1.62 | 5.06 | 3.35 | 1.86 | 1.99 | 1.25 |

Cells which migrated to the lower surface of the Matrigel-coated filter in the Boyden chamber were counted after 24-h incubation. Kuramochi+anti-HGF represents the data with 500 ng ml$^{-1}$ of anti-HGF antibody in both upper and lower chambers. Values are means ± s.d. from triplicate determinations.

**Table 2** Checkerboard analysis of Kuramochi cells

| | | Upper chamber | | |
|---|---|---|---|---|
| | | 0 ng ml$^{-1}$ | 1 ng ml$^{-1}$ | 10 ng ml$^{-1}$ |
| Lower chamber | 0 ng ml$^{-1}$ | 69 ± 10 | 226 ± 57 | 90 ± 36 |
| | 1 ng ml$^{-1}$ | 196 ± 100 | 333 ± 64 | 405 ± 96 |
| | 10 ng ml$^{-1}$ | 404 ± 65 | 583 ± 46 | 665 ± 106 |

Various concentrations of HGF were added into the upper and/or lower compartments. The cells which migrated to the lower surface of the filter of the Boyden chamber were counted after 10-h incubation. Values are means ± s.d. from triplicate determinations.

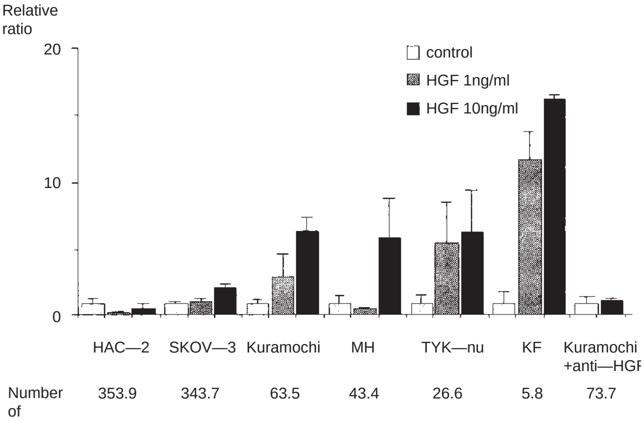

**Figure 3** Cell motility of ovarian cancer cells. A total of 1 ng ml$^{-1}$ or 10 ng ml$^{-1}$ of HGF was added into the lower compartments of the Boyden chambers. Migrated cells were determined as described in Materials and Methods. Values are means ± s.d. from triplicate determinations. Number of control represent numbers of migrated cells in the absence of HGF, and the bars represent the relative ratio to the control. The motility of each cell line was enhanced by treatment with HGF. Kuramochi + anti-HGF represents the data with 500 ng ml$^{-1}$ of anti-HGF antibody in both upper and lower compartments of the chambers. Addition of anti-HGF antibody abrogated HGF induced cell motility involving a single base substitution at codon 12 of K-*ras*, changing a glycine to valine in MCAS, and at N-*ras* codon 12 causing substitution of aspartic acid for glycine in PA-1. Thus, we excluded these two cell lines from further analysis. No mutations were detected at codon 12, 13 or 61 of the K-, H- or N-*ras* genes in the remaining six cancer cell lines.

There is considerable evidence that Ras proteins are critical relay switches that control signalling pathways connecting the cell surface with the nucleus. Activated GTP-bound Ras has the potential to phosphorylate and activate a cascade of serine/threonine kinases. Thus, we investigated the alterations in MAPK activity in response to HGF (Figure 2B). Migration of the active form of MAPK was slower than that of the inactive MAPK form, resulting in an electrophoretic mobility shift of the 42 kDa protein. HGF treatment resulted in a marked increase in the level of active MAPK in ovarian cancer cell lines. This was supported by the definite signal reaction with phospho-specific MAPK antibody. In contrast, inactive, unphosphorylated MAPK was predominant in the absence of HGF. Our results indicated that HGF caused HGFR phosphorylation and MAPK activation.

### HGF-stimulated motogenic and invasive activity of ovarian cancer cells

Next, we examined the effects of HGF on the motogenic activity of the panel of six ovarian cancer cell lines (Figure 3). The cells which migrated to the lower surface of the filter were counted after 10 h incubation. We used the conditioned medium from NIH3T3 cells to stimulate the basal level of motogenic activity of the cells. To address whether the promotion of motogenic activity is specific to human HGF, we investigated the alteration in motility and invasiveness of Kuramochi cells in the presence or the absence of 500 ng ml$^{-1}$ anti-human HGF antibody. Basal levels of motility and invasiveness were not affected by the anti-HGF antibody treatment in Kuramochi cells (Figure 3 and Table 1). The antibody-treated Kuramochi cells stimulated with HGF corresponded to the suppression of cell migration and invasiveness to the constitutive level, whereas the antibody-non-treated Kuramochi cells responded to HGF and markedly enhanced their motility and invasiveness. Thus, the data demonstrated the specific human HGF effect on the cell motility and invasiveness of ovarian cancer cells.

The motility of four cell lines (Kuramochi, MH, TYK-nu and KF) was enhanced 6–16 times by treatment with HGF in a dose-dependent manner. However, the promotion of cell motility was unremarkable in SKOV-3 and HAC-2 cells, which showed relatively high constitutive motility. To determine whether the

© 2000 Cancer Research Campaign

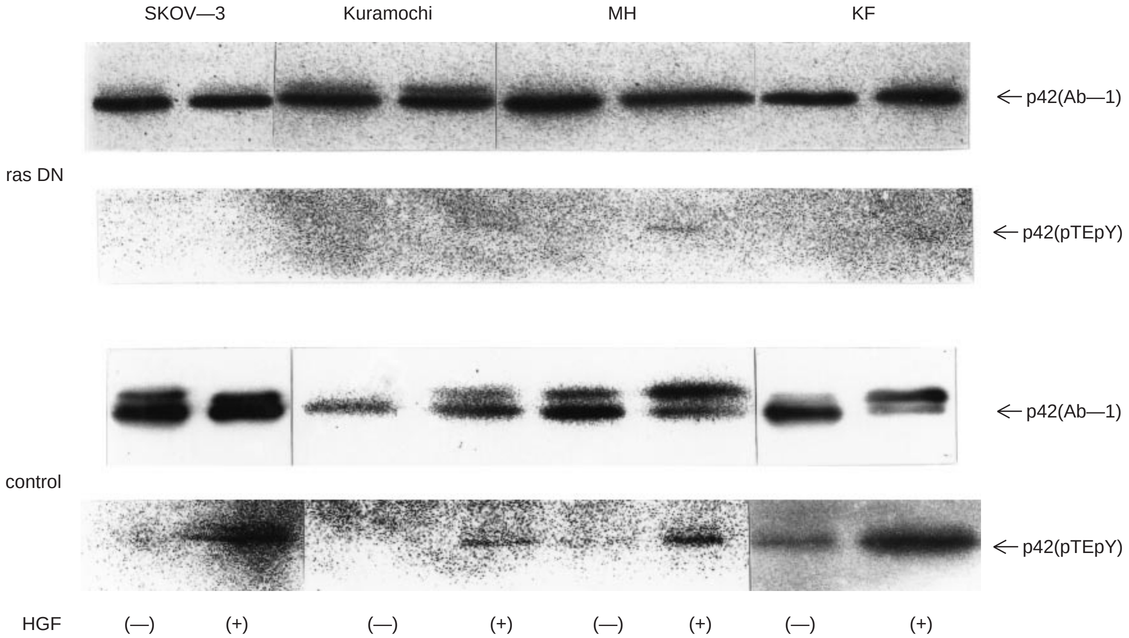

**Figure 4** Effects of dominant-negative Ras on the phosphorylation of p42 MAPK. Western blotting analysis of four cell lines infected with AdexCAHRasY57. Twenty-four hours after transfection with the adenoviral vector, the medium was replaced with serum-free RPMI for 48 h and 10 ng $ml^{-1}$ HGF was added for 15 min. Cells were lysed and MAPK activation was determined with anti-MAPK monoclonal antibody (Ab-1) and anti-phospho-specific MAPK antibody (pTEpY). Activation of MAPK by HGF treatment was suppressed in all ovarian cancer cell lines expressing the dominant-negative Ras (*ras* DN). The lower lanes show cells infected with AdCALacZ virus (control). The arrows indicate p42 MAPK of which the activation is shown by a band shift (Ab-1) or reaction with pTEpY

motogenic stimulation by HGF was due to either enhanced chemokinetic or chemotactic activity, we performed the checkerboard assay repeatedly using Kuramochi cells that were sensitive to HGF (Table 2). A larger effect was obtained when HGF was added into the lower compartment only, indicating that HGF exerted a dose-dependent chemotactic effect. Addition of HGF (10 ng $ml^{-1}$) to both compartments induced a 9.6-fold stimulation of migration compared with the value obtained without addition of HGF. These results presumably represented the general response of cell motility induced by HGF.

The responsiveness of the cell lines was further examined by the invasion assay in which the cells were plated on Matrigel-coated filters for 24 h (Table 1). In this assay, SKOV-3 cells showed a higher basal level of invasiveness compared with the other cells. In the presence of HGF, the invasive activity was augumented in five of six cell lines; 1.62-fold in SKOV-3, 5.06-fold in Kuramochi, 3.35-fold in MH, 1.86-fold in TYK-nu, and 1.99-fold in KF. HAC-2 cells showed no increase in invasive activity in response to HGF. These observations were still compatible with the notion that HGF mediated the motogenic signal through its receptor, although there were cell type-specific variations in migration and invasiveness.

### Regulation of HGF-mediated stimulation by the Ras-dependent signalling

The present study demonstrated a positive correlation between MAPK activation induced by HGF and enhanced cell migration and the resultant increase in invasiveness in the ovarian cancer cell lines. To determine directly whether the Ras-dependent signalling pathway is required for cancer cell migration and invasiveness, we infected the four ovarian cancer cell lines, SKOV-3, Kuramochi, MH and KF, with the recombinant adenovirus AdexCAHRasY57 expressing the dominant-negative Ras Y57 mutant protein. This mutant protein has been shown to interfere with the function of endogenous Ras (Ueno et al, 1997). We determined the infection efficiency by co-infecting with AdexCAHRasY57 and AdCALacZ. After 72-h incubation of co-infection, the percentage of cells stained positively for β-galactosidase was counted. The percentages of positively stained cells were 99.2% in SKOV-3, 99.4% in KF, 97.0% in MH and 95.8% in Kuramochi cells respectively.

The Ras-MAPK cascade plays an important role in cell proliferation. Therefore, we analysed the influence of dominant-negative Ras Y57 mutant protein on cell growth. Cell death was undetectable within the 120-h incubation following infection of these four ovarian cancer cell lines with AdexCAHRasY57. As expected, Ras Y57 interfered with growth of the four ovarian cancer cells. The relative cell number at the end of the 120-h incubation period of the infected ovarian cancer cells ranged from 21.6% to 56.8%, as compared with uninfected cell cultures. However, cell growth suppression due to Ras Y57 expression was undetectable following 24-h incubation after infection. The relative cell number of infected/uninfected cells was 96.9% in SKOV-3, 86.7% in Kuramochi, 97.6% in MH and 98.8% in KF. Thus, the effects of dominant-negative Ras Y57 on the motility and invasiveness of four ovarian cancer cell lines was evaluated with observation periods of 10 h and 24 h respectively.

Ras Y57 completely abrogated MAPK activation in response to HGF in these four ovarian cancer cell lines, which was demonstrated by using the infected cells incubated for 48 h with serum-free media followed by HGF stimulation for 15 min (Figure 4). We thus tested whether the extinction of HGF-stimulated MAPK activation resulted in suppression of ovarian cancer cell migration (Table 3). Twenty-four hours after AdexCAHRasY57 infection,

 *© 2000 Cancer Research Campaign*

**Table 3** Motility assay

| Cell line | | SKOV-3 | Kuramochi | MH | KF |
|---|---|---|---|---|---|
| Control | HGF (–) | 135.2 ± 16.0 | 88.3 ± 36.8 | 51.5 ± 20.5 | 5.0 ± 2.9 |
| | HGF (+) | 401.1 ± 83.3 | 391.2 ± 81.2 | 252.1 ± 129.3 | 65.3 ± 10.2 |
| *ras* DN | HGF (–) | 97.5 ± 36.0 (72.1%) | 26.7 ± 7.9 (30.2%) | 5.7 ± 3.6 (11.0%) | 1.3 ± 0.9 (26.0%) |
| | HGF (+) | 223.3 ± 78.6 (55.7%) | 212.7 ± 20.0 (54.4%) | 151.8 ± 41.5 (60.2%) | 6.0 ± 3.3 (9.2%) |

Control and ras DN represent cells infected with control virus AdCALacZ and dominant-negative Ras virus AdexCAHRasY57 respectively. A total of 10 ng $ml^{-1}$ of HGF were added into the lower compartment of the Boyden chamber and cells were incubated for 10 h. The ratios of the migrated cell number compared to control virus-infected cells are shown in parentheses.

**Table 4** Invasion assay

| Cell line | | SKOV-3 | Kuramochi | MH | KF |
|---|---|---|---|---|---|
| Control | HGF (–) | 1346.0 ± 215.5 | 167.3 ± 25.7 | 170.0 ± 92.6 | 460.0 ± 76.5 |
| | HGF (+) | 2323.8 ± 89.5 | 563.3 ± 153.1 | 604.3 ± 41.3 | 947.2 ± 170.3 |
| *ras* DN | HGF (–) | 109.1 ± 13.7 (8.1%) | 26.0 ± 13.7 (15.6%) | 4.3 ± 2.6 (2.5%) | 3.7 ± 3.8 (0.7%) |
| | HGF (+) | 92.3 ± 18.7 (4.0%) | 43.0 ± 20.8 (7.6%) | 29.7 ± 16.2 (4.9%) | 28.0 ± 33.4 (3.0%) |

A total of 10 ng/ml of HGF was added into the upper and lower compartments of the Boyden chamber, and cells were incubated for 24 h. The ratios of the migrated cell number compared to control virus-infected cells are shown in parentheses.

$5 \times 10^4$ cells were plated in the upper compartment of Boyden chambers. The number of cells which migrated to the lower surface was counted after 10 h incubation. The HGF-stimulated cell migration was most clearly inhibited in KF cells following AdexCAHRasY57 infection (Table 3). Ras Y57-expressed KF cells stimulated with HGF corresponded to the suppression of cell migration to the constitutive level. These results indicated that HGF acted on cell motility through a Ras-dependent pathway in KF cells. In turn, partial suppression of HGF-stimulated cell migration was observed in SKOV-3 (55.7%), Kuramochi (54.4%) and MH (60.2%) cells. Thus, it is possibile that the motogenic response of ovarian cancer cells to HGF involves Ras-mediated signal transduction but its importance is dependent on the cell type.

We then examined the alterations in invasiveness of the ovarian cancer cells following infection with AdexCAHRasY57 (Table 4). Twenty-four hours after infection, $1 \times 10^5$ cells were plated in Boyden chambers. The potential of HGF to stimulate invasive activity in response to HGF was completely abrogated in all of the ovarian cancer cells infected with AdexCAHRasY57.

### Regulation of HGF-mediated responses by downstream effectors of Ras

It is now evident that the altered responses of ovarian cancer cells brought about by HGF-mediated signalling are Ras-dependent, and the events occurring downstream of this protein are the subject of investigation. Ras activates a number of signalling pathways through its ability to activate key effector proteins. The most extensively studied of these are the MAP kinase pathway and the PI3-kinase pathway (Webb et al, 1998; Potempa et al, 1998). Thus, we evaluated the involvement of these pathways in the motogenic activity of ovation cancer cells. We used PD098059 as a MEK inhibitor and LY294002 as a PI3-kinase inhibitor, respectively, to abrogate signal transduction through each pathway. First of all, we examined the effect of each inhibitor (10 μM PD098059 and 50 μM LY294002) on Kuramochi cell growth as signal transduction through the MAPK and the PI3-kinase cascades is involved in cell proliferation. However, a 24 h treatment of Kuramochi cells either with PD098059 or LY294002 did not result in cell growth inhibition, compared with the DMSO-treated control cells (data not shown).

HGF treatment resulted in a marked increase in the level of active Akt protein that was a downstream effector of PI3-kinase, and phosphorylated MAPK in the absence of inhibitors. In contrast, inactive, unphosphorylated MAPK or Akt was predominant in PD098059-treated or LY294002-treated Kuramochi cells even in the presence of HGF. The level of active MAPK and Akt was almost 20% and 10% of that in the HGF-stimulated control (data not shown). The significant reduction in active MAPK by the MEK inhibitor resulted in the suppression of constitutive motility (59.9% of that in DMSO-treated cells) (Figure 5). However, the stimulation of cell motility in response to HGF was retained in PD098059-treated cells. In turn, PD098059 did not exhibit any inhibitory effects on the constitutive invasiveness of Kuramochi cells, but its stimulation in response to HGF was completely abrogated. In contrast with PD098059, a PI3-kinase inhibitor, LY294002 markedly inhibited the constitutive motility of Kuramochi cells (14% of that in DMSO-treated control cells). Again, stimulation of cell motility in response to HGF was exhibited in LY294002-treated Kuramochi cells. None of the inhibitory effects on constitutive invasiveness were shown by LY294002 treatment but the stimulation of invasiveness in response to HGF was abrogated in LY294002-treated Kuramochi cells. The results

© 2000 Cancer Research Campaign 

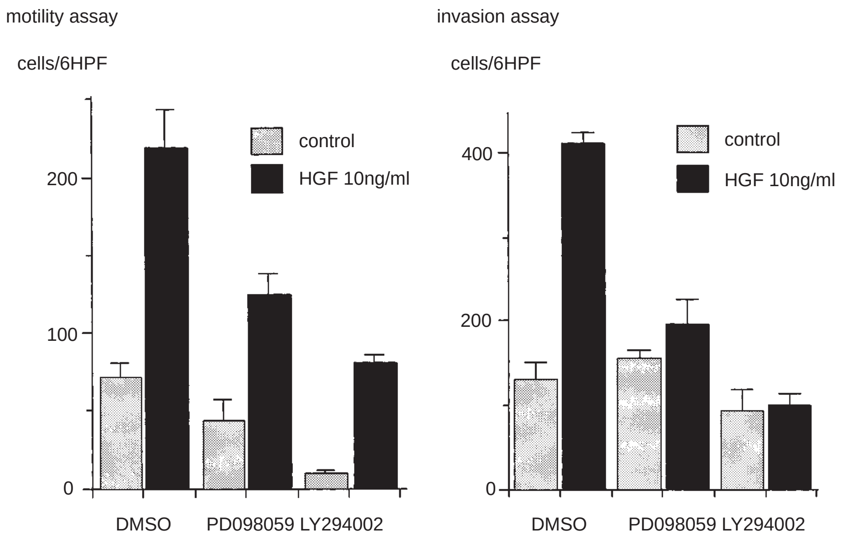

**Figure 5** Effects of MEK inhibitor and PI3-kinase inhibitor on cell motility and invasion. Kuramochi cells were plated in Boyden chambers in the presence of 10 μM PD098059 or 50 μM LY294002. Cells were treated with the same amount of carrier (DMSO) as the control. Motility and invasion were assayed as described in Materials and Methods. Both PD098059 and LY294002 abrogated the HGF-stimulated invasiveness of Kuramochi cells, whereas their inhibitory effect on the cell motility was not seen suggested the implication of both MAPK and PI3-kinase pathways in HGF-stimulated invasiveness of ovarian cancer cells. In contrast, HGF-stimulated promotion of Kuramochi cell motility was seemingly independent of the MAPK and PI3-kinase pathways.

### DISCUSSION

The present study demonstrated the overexpression of HGFR in ovarian cancer cells, although there was considerable variation in the HGFR level among individual cell lines. Overexpressed HGFR had the potential to promote its autophosphorylation followed by MAPK activation in response to HGF. MAPK activation may be induced by activated Ras, indicating that the ovarian cancer cell lines conserve the signalling pathway via Ras protein as a downstream target of HGFR. In addition, we found no evidence for the HGF-HGFR autocrine loop in these cells.

Concomitant with MAPK activation, HGF promoted the motility of ovarian cancer cells. Cell motility was markedly enhanced in the cell lines with relatively low constitutive motility. In contrast, those cells with high constitutive motility were relatively insensitive to HGF, suggesting that the constitutive motility is the cumulative effect of signalling pathways unrelated to the HGF-HGFR system. Activation of these signalling pathways in the cells would mask the HGF-mediated stimulation of cell motility. The stimulatory effect of HGF was blocked completely in KF cells and partially in the remaining cells by the dominant-negative Ras Y57 mutant protein. These results suggest that Ras-dependent signalling is a major route of HGF-mediated stimulation of cell motility. However, Ras-independent signalling was also involved, at least partially, in the motility activation of ovarian cancer cells in response to HGF. Thus, the present findings suggest that the induction of cell motility is a major aspect of the action of HGF that is transmitted through the Ras-dependent cascade in ovarian cancer cells. The *ras* mutations detected in almost 30% of ovarian cancers would correspond to the constitutive enhancement of signalling for cell motility.

The motility signal of HGF is transduced further downstream of Ras. Ras is known to activate multiple signal transduction pathways, including the MAPK cascade, and PI3-kinase cascade. By the use of a specific MEK inhibitor (PD098059) or a PI3-kinase inhibitor (LY294002), we showed that seemingly neither the MAPK nor the PI3-kinases pathway was essential to the HGF-induced motility of Kuramochi cells. However, the stimulatory effect of HGF exhibited by the dominant-negative Ras Y57-infected Kuramochi cells indicates the presence of Ras-independent signalling; an eightfold enhancement of cell motility was shown in these cells. This Ras-independent signalling in response to HGF would mask the inhibitory effect of PD098059 or LY294002. As it has been shown that HGF activates MAPK and PI3-kinase, and PI3-kinase in turn leads to activation of Rac (Parker et al, 1995), further detailed studies are now required to address whether the MAPK and PI3-kinase cascades are involved in cell motility of ovarian cancer cells.

Another intriguing property of HGF in ovarian cancer cells is its ability to promote invasion of the extracellular matrix. Similarly to its effect on cell motility, HGF showed the potential to induce invasion concomitant with MAPK activation, and expression of dominant-negative Ras Y57 completely inhibited the HGF-induced invasiveness of ovarian cancer cells. These observations suggest that the HGF-HGFR-Ras signalling cascade is involved importantly in the invasive phenotype shown by ovarian cancer cells. As expected from the dominant-negative Ras Y57 infection, both MEK and PI3-kinase inhibitors completely abrogated the stimulation of invasiveness in response to HGF. These results are compatible with the supposition that HGF-stimulated ovarian

*© 2000 Cancer Research Campaign*

cancer cell invasiveness requires the MAPK and PI3-kinase pathways downstream of Ras. Tumour cell invasion is the end result of a complex series of steps. Cancer cells must move through the extracellular matrix during invasion. Cell migration is considered to be a key component of tumour cell invasion. However, the extent of contribution of Ras-dependent signalling in response to HGF is not identical between cell motility and invasiveness of Kuramochi cells. HGF-HGFR signalling is coupled to the activation of proteases that mediate degradation of the extracellular matrix-basement membrane, and play an impotent role in cellular invasion and metastasis (Jeffer et al, 1996*b*). Thus, intensive studies focusing on the interaction between the protease activation and Ras-dependent signalling are now in progress.

### REFERENCES

Boccacino C, Ando M, Tamagnone L, Bardelli A, Michieli P, Battistini C and Comoglio PM (1998) Induction of epithelial tubules by growth factor HGF depends on the STAT pathway. *Nature* **391**: 285–288

Corps AN, Sowter HM and Smith SK (1997) Hepatocyte growth factor stimulates motility, chemotaxis and mitogenesis in ovarian carcinoma cells expressing high levels of c-Met. *Int J Cancer* **73**: 151–155

DiRenzo MF, Olivero M, Katsaros D, Crepaldi T, Gaglis P, Zola P, Sismondi P and Comoglio PM (1994) Overexpression of the Met/HGF receptor in ovarian cancer. *Int J Cancer* **58**: 658–662

Hartmann G, Weidner KM, Schwartz H and Birchmeier W (1994) The motility signal of scatter factor/hepatocute growth factor mediated through the receptor tyrosine kinase Met requires intracellular action of Ras. *J Biol Chem* **269**: 21936–21939

Jeffer M, Rong S, Anver M and VandeWoude GF (1996*a*) Autocrine hepatocyte growth favtor/scatter factor-met signaling induces transformation and invasive/metastatic phenotype in C127 cells. *Oncogene* **13**: 853–861

Jeffer M, Rong S and VandeWoude GF (1996*b*) Enhanced tumorigenecity and invasion-metastasis by hepatocyte growth factor/scatter factor-met signaling in human cells concomitant with induction of the urokinase proteolysis network. *Mol Cel Biol* **16**: 1115–1125

Kobayashi H, Moniwa N, Gotoh J, Sugimura M and Terao T (1994) Role of activated protein C in facilitating basement membrane invasion by tumor cells. *Cancer Res* **54**: 261–267

Lamszus K, Schmidt NO, Jin L, Laterra J, Zagzag D, Way D, White M, Weinand M, Goldberg ID, Westphal M and Rosen EM (1998) Scatter factor promotes motility of human glioma and neuromicrovascular endotherial cells. *Int J Cancer* **75**: 19–28

Moghul A, Lin L, Beedle A, Kanbour-Shakir A, DeFrances MC, Liu Y and Zarnegar R (1994) Modulation of c-MET proto-oncogene (HGF receptor) mRNA abundance by cytokines and hormones: evidence for rapid decay of 8 kb c-MET transcript. *Oncogene* **9**: 2045–2052

Montesano R, Matsumoto K, Nakamura T and Orci L (1991) Identification of a fibroblast-derived epithelial morphoge as hepatocyte growth factor. *Cell* **67**: 901–908

Nakamura T, Matsumoto K, Kiritoshi A, Tano Y and Nakamura T (1997) Induction of hepatocyte growth factor in fibroblasts by tumor-derived factors affects invasive growth of tumor cells: in vitro analysis of tumor–stromal interactions. *Cancer Res* **57**: 3305–3313

Parker PJ (1995) Intracellular signaling. PI 3-kinase puts GTP on the rac. *Curr Biol* **5**: 577–579

Pelicci G, Giordano S, Zhen Z, Salcini AE, Lanfrancone L, Bardelli A, Panayotou G, Waterfield MD, Ponzetto C, Pelicci PG and Comoglio PM (1995) The motogenic and mitogenic responses to HGF are amplified by the Shc adaptor protein. *Oncogene* **10**: 1631–1638

Ponzetto C, Bardelli A, Zhen Z, Maina F, Zonca PD, Giordano S, Graziani A, Panayotou G and Comoglio PM (1994) A multifunctional docking site mediates signaling and transformation by the hepatocyte growth factor/scatter factor receptor family. *Cell* **77**: 261–271

Potempa S and Ridley AJ (1998) Activation of both MAP kinase and phosphatidylinositide 3-kinase by ras is required for hepatocyte growth factor/scatter factor-induced adherens junction disassembly. *Mol Cell Biol* **9**: 2185–2200

Ridley AJ, Comoglio PM and Hall A (1995) Regulation of scatter factor/hepatocyte growth factor responses by ras, rac, and rho in MDCK cells. *Mol Cell Biol* **15**: 1110–1122

Rosen Em, Nigam SK and Goldberg ID (1994) Scatter factor and the c-Met receptor: a paradigm for mesenchymal/epitherial interaction. *J Cell Biol* **127**: 1783–1787

Stoker M, Gherardi E, Perryman M and Gray J (1987) Scatter factor is a fibroblast-derived modulator of epithelial cell motility. *Nature* **327**: 239–242

Teneriello MG, Ebina M, Linnoila RI, Henry M, Nash JD, Park RC and Bierrer MJ (1993) p53 and Ki-ras gene mutations in epithelial ovarian neoplasms. *Cancer Research* **53**: 3103–3108

Ueno H, Yamamoto H, Ito S, Li JJ and Takeshita A (1997) Adenovirus-mediated transfer of a dominant-negative H-ras suppresses neointimal formation in balloon-injured arteries in vivo. *Arterioscler Thromb Vasc Biol* **17**: 898–904

Webb CP, Aelst LV, Wilger MH and VandeWoude GF (1998) Signaling pathways in ras-mediated tumorigenicity and metastasis. *Proc Natl Acad Sci USA* **95**: 8773–8778

© 2000 Cancer Research Campaign

## References

[PDF_00764] Boccaccio C., Andò M., Tamagnone L., Bardelli A., Michieli P., Battistini C., Comoglio P. M. (1998). Induction of epithelial tubules by growth factor HGF depends on the STAT pathway.. Nature.

[PDF_00767] Corps A. N., Sowter H. M., Smith S. K. (1997). Hepatocyte growth factor stimulates motility, chemotaxis and mitogenesis in ovarian carcinoma cells expressing high levels of c-met.. Int J Cancer.

[PDF_00770] Di Renzo M. F., Olivero M., Katsaros D., Crepaldi T., Gaglia P., Zola P., Sismondi P., Comoglio P. M. (1994). Overexpression of the Met/HGF receptor in ovarian cancer.. Int J Cancer.

[PDF_00773] Hartmann G., Weidner K. M., Schwarz H., Birchmeier W. (1994). The motility signal of scatter factor/hepatocyte growth factor mediated through the receptor tyrosine kinase met requires intracellular action of Ras.. J Biol Chem.

[PDF_00776] Jeffers M., Rong S., Anver M., Vande Woude G. F. (1996). Autocrine hepatocyte growth factor/scatter factor-Met signaling induces transformation and the invasive/metastastic phenotype in C127 cells.. Oncogene.

[PDF_00779] Jeffers M., Rong S., Vande Woude G. F. (1996). Enhanced tumorigenicity and invasion-metastasis by hepatocyte growth factor/scatter factor-met signalling in human cells concomitant with induction of the urokinase proteolysis network.. Mol Cell Biol.

[PDF_00783] Kobayashi H., Moniwa N., Gotoh J., Sugimura M., Terao T. (1994). Role of activated protein C in facilitating basement membrane invasion by tumor cells.. Cancer Res.

[PDF_00786] Lamszus K., Schmidt N. O., Jin L., Laterra J., Zagzag D., Way D., Witte M., Weinand M., Goldberg I. D., Westphal M. (1998). Scatter factor promotes motility of human glioma and neuromicrovascular endothelial cells.. Int J Cancer.

[PDF_00790] Moghul A., Lin L., Beedle A., Kanbour-Shakir A., DeFrances M. C., Liu Y., Zarnegar R. (1994). Modulation of c-MET proto-oncogene (HGF receptor) mRNA abundance by cytokines and hormones: evidence for rapid decay of the 8 kb c-MET transcript.. Oncogene.

[PDF_00794] Montesano R., Matsumoto K., Nakamura T., Orci L. (1991). Identification of a fibroblast-derived epithelial morphogen as hepatocyte growth factor.. Cell.

[PDF_00797] Nakamura T., Matsumoto K., Kiritoshi A., Tano Y., Nakamura T. (1997). Induction of hepatocyte growth factor in fibroblasts by tumor-derived factors affects invasive growth of tumor cells: in vitro analysis of tumor-stromal interactions.. Cancer Res.

[PDF_00801] Parker P. J. (1995). Intracellular signalling. PI 3-kinase puts GTP on the Rac.. Curr Biol.

[PDF_00803] Pelicci G., Giordano S., Zhen Z., Salcini A. E., Lanfrancone L., Bardelli A., Panayotou G., Waterfield M. D., Ponzetto C., Pelicci P. G. (1995). The motogenic and mitogenic responses to HGF are amplified by the Shc adaptor protein.. Oncogene.

[PDF_00807] Ponzetto C., Bardelli A., Zhen Z., Maina F., dalla Zonca P., Giordano S., Graziani A., Panayotou G., Comoglio P. M. (1994). A multifunctional docking site mediates signaling and transformation by the hepatocyte growth factor/scatter factor receptor family.. Cell.

[PDF_00811] Potempa S., Ridley A. J. (1998). Activation of both MAP kinase and phosphatidylinositide 3-kinase by Ras is required for hepatocyte growth factor/scatter factor-induced adherens junction disassembly.. Mol Biol Cell.

[PDF_00815] Ridley A. J., Comoglio P. M., Hall A. (1995). Regulation of scatter factor/hepatocyte growth factor responses by Ras, Rac, and Rho in MDCK cells.. Mol Cell Biol.

[PDF_00818] Rosen E. M., Nigam S. K., Goldberg I. D. (1994). Scatter factor and the c-met receptor: a paradigm for mesenchymal/epithelial interaction.. J Cell Biol.

[PDF_00821] Stoker M., Gherardi E., Perryman M., Gray J. (1987). Scatter factor is a fibroblast-derived modulator of epithelial cell mobility.. Nature.

[PDF_00823] Teneriello M. G., Ebina M., Linnoila R. I., Henry M., Nash J. D., Park R. C., Birrer M. J. (1993). p53 and Ki-ras gene mutations in epithelial ovarian neoplasms.. Cancer Res.

[PDF_00826] Ueno H., Yamamoto H., Ito S., Li J. J., Takeshita A. (1997). Adenovirus-mediated transfer of a dominant-negative H-ras suppresses neointimal formation in balloon-injured arteries in vivo.. Arterioscler Thromb Vasc Biol.

[PDF_00830] Webb C. P., Van Aelst L., Wigler M. H., Vande Woude G. F. (1998). Signaling pathways in Ras-mediated tumorigenicity and metastasis.. Proc Natl Acad Sci U S A.

